# Utilizing Immuno-Oncology registry data for enhanced non-small cell lung cancer treatment predictions

**DOI:** 10.1093/jamiaopen/ooaf069

**Published:** 2025-07-09

**Authors:** Yili Zhang, Shaked Lev-Ari, Jacob Zaemes, Alexandra Della Pia, Bianca DeAgresta, Samir Gupta, Alex Marki, Rachel Zemel, Andrew Ip, Adil Alaoui, Charalampos Charalampous, Iris Rahman, Olivia Wilkins, Subha Madhavan, Peter McGarvey, Lauren Pascual, Michael B Atkins, Neil J Shah

**Affiliations:** Innovation Center for Biomedical Informatics, Georgetown University, Washington, DC, 20007, United States; Ella Lemelbaum Institute for Immuno-Oncology, Sheba Medical Center at Tel Hashomer, Ramat Gan, 526260, Israel; Harvard Medical Faculty Physicians, Beth Israel Deaconess Medical Center Inc, Boston, MA, 02215, United States; John Theurer Cancer Center, Hackensack Meridian Health, Hackensack, NJ, 07061, United States; John Theurer Cancer Center, Hackensack Meridian Health, Hackensack, NJ, 07061, United States; Innovation Center for Biomedical Informatics, Georgetown University, Washington, DC, 20007, United States; MedStar Georgetown University Hospital, Washington, DC, 20007, United States; MedStar Georgetown University Hospital, Washington, DC, 20007, United States; John Theurer Cancer Center, Hackensack Meridian Health, Hackensack, NJ, 07061, United States; Innovation Center for Biomedical Informatics, Georgetown University, Washington, DC, 20007, United States; MedStar Georgetown University Hospital, Washington, DC, 20007, United States; MedStar Washington Hospital Center, Washington, DC, 20010, United States; MedStar Georgetown University Hospital, Washington, DC, 20007, United States; Innovation Center for Biomedical Informatics, Georgetown University, Washington, DC, 20007, United States; Pfizer Research and Development, New York, NY, 11206, United States; Innovation Center for Biomedical Informatics, Georgetown University, Washington, DC, 20007, United States; John Theurer Cancer Center, Hackensack Meridian Health, Hackensack, NJ, 07061, United States; Georgetown-Lombardi Comprehensive Cancer Center, Georgetown University Medical Center, Washington, DC, 20007, United States; Department of Medicine, Memorial Sloan Kettering Cancer Center, New York, NY, 10065, United States

**Keywords:** NSCLC, immunotherapy, treatment response, prediction model, clinical data

## Abstract

**Objectives:**

We aim to leverage more comprehensive phenotypic and genotypic clinical data to enhance the treatment response predictions.

**Materials and Methods:**

The study cohort includes 213 NSCLC patients who underwent ICI therapy. Patients were categorized based on treatment outcomes: those with complete or partial responses were considered responders, while those exhibiting stable or progressive disease were deemed non-responders. Comprehensive phenotypic and genomic features were selected for prediction. We developed 9 machine learning models. The model demonstrating the highest area under the receiver operating characteristic curve (AUROC) performance was further analyzed using Shapley additive explanation values to interpret the predictive factors.

**Results:**

There were 72 patients who responded to the treatment, while 141 patients were considered non-responders. In total, 57 features were included, encompassing demographics, tumor status, treatment information, pre-treatment information, serum CBC, serum chemistry, and vital signs. The KNN model excelled among the models, achieving an AUROC score of 0.862 and outperforming the conventional PD-L1 biomarker’s AUROC of 0.619. The top features influencing ICI treatment response include the ECOG performance status of 0, lower red cell distribution width, higher mean platelet volume, etc.

**Discussion:**

The significance of functional status, inflammatory biomarkers, and PD-L1 expression are revealed. This research underscores the potential of using a more nuanced combination of biochemical markers and clinical data to enhance the precision of immunotherapy efficacy predictions, compared with single prognostic biomarkers such as PD-L1.

**Conclusion:**

Our findings emphasize the complex interplay among various risk factors that influence the effectiveness of ICI.

## Introduction

Lung cancer is the leading cause of cancer-related deaths in the United States, with non-small cell lung cancer (NSCLC) constituting the most common form. The widespread occurrence of NSCLC and its high case fatality rate have highlighted the critical need for novel and more effective treatment strategies. In recent years, the significant advancement of immunotherapy has provided new treatment options to patients battling this disease and even a chance to achieve prolonged complete remission. Immune checkpoint inhibitors (ICIs) unleash the power of the immune system to battle cancer and have become a critical component of cancer immunotherapy.^1^ With the advent of ICIs, such as pembrolizumab and nivolumab, clinical trials and real-world data have consistently shown that immunotherapy can extend survival, enhance the quality of life, and provide long-lasting responses in patients with metastatic NSCLC.^2^ Nonetheless, treatment outcomes may differ significantly among individuals with some experiencing substantial and lasting remissions, while others deriving minimal benefit or quickly developing resistance to the therapy.^3^ To measure treatment efficacy, clinicians and researchers traditionally rely on standard response criteria, such as the Response Evaluation Criteria in Solid Tumors version 1.1 (RECIST 1.1) classifications,^4^ where treatment responses are classified at predefined time points into categories such as complete response (CR), partial response (PR), progressive disease (PD), and stable disease (SD). A response to ICI is typically more durable frequently being sustained in response assessments performed every 6-12 weeks. However, the disparity in treatment outcomes poses a complex challenge for oncologists, emphasizing the need for precise and dependable methods to forecast individual patient responses to immunotherapy before treatment initiation.

Traditionally, biomarkers such as programmed death-ligand 1 (PD-L1), tumor mutational burden (TMB), and specific genomic alterations have emerged as significant predictors of immunotherapy outcomes.^5^ However, due to the variable and dynamic nature of tumor expression of these markers, these markers provide only a partial prediction of treatment response. For instance, patients with low PD-L1 expression may still derive benefit from immunotherapy, while those with high expression levels may exhibit resistance.^6^ The absence of standardized threshold values for high TMB or PD-L1 further complicates them as predictive markers. Previous research focusing on the prediction of response to immunotherapy has predominantly centered on gene expression profiles^7^^,^^8^ and immunohistochemical or immunofluorescent imaging data.^9^ However, these data types, along with PD-L1 and TMB metrics, involve complex collection methods, varied sample sources, prolonged processing times, and associated costs and accessibility issues.

Compared to genetic data points, clinical and phenotypic information provides observable outcomes, offers a more holistic view of a patient’s health, and is more readily accessible during routine clinical evaluations. This phenotypic data include prior treatment responses, present health status, and organ function indicated by blood testing, as well as lifestyle indicators such as smoking history. Incorporating phenotypic data enables a prediction model to capture the nuanced dynamics of immune responses and the intricate interactions between the tumor and the host’s biological systems. Adopting both genotype and phenotype data could yield a more extensive and individualized prediction of immunotherapy response, thus improving treatment customization for each patient, advancing healthcare outcomes, and increasing therapy effectiveness. Machine learning has become an invaluable tool in multifactorial clinical decision-making.^10^ Its ability to reveal complex spatial patterns and analyze diverse medical datasets has led to the discovery of novel insights and predictive factors that inform treatment strategies. Notable examples include the use of machine learning to analyze radiological images for early cancer detection,^11^ optimize radiation therapy planning,^12^ and predict chemotherapy response.^13^

Consequently, this paper seeks to delve into the complexities of NSCLC immunotherapy and the role of machine learning in utilizing phenotypic and genotypic information to predict outcomes prior to treatment initiation. There are 2 objectives: (1) to develop a machine learning model that leverages comprehensive patient phenotypic and genotypic data before treatment to predict treatment response, and (2) to ascertain key biomarkers within the predictive model, which may serve as potential critical factors in treatment response. This research endeavors to provide a roadmap for the future of NSCLC treatment, where personalized and precise immunotherapy yields better cancer care outcomes.

## Methods

**Figure 1.**
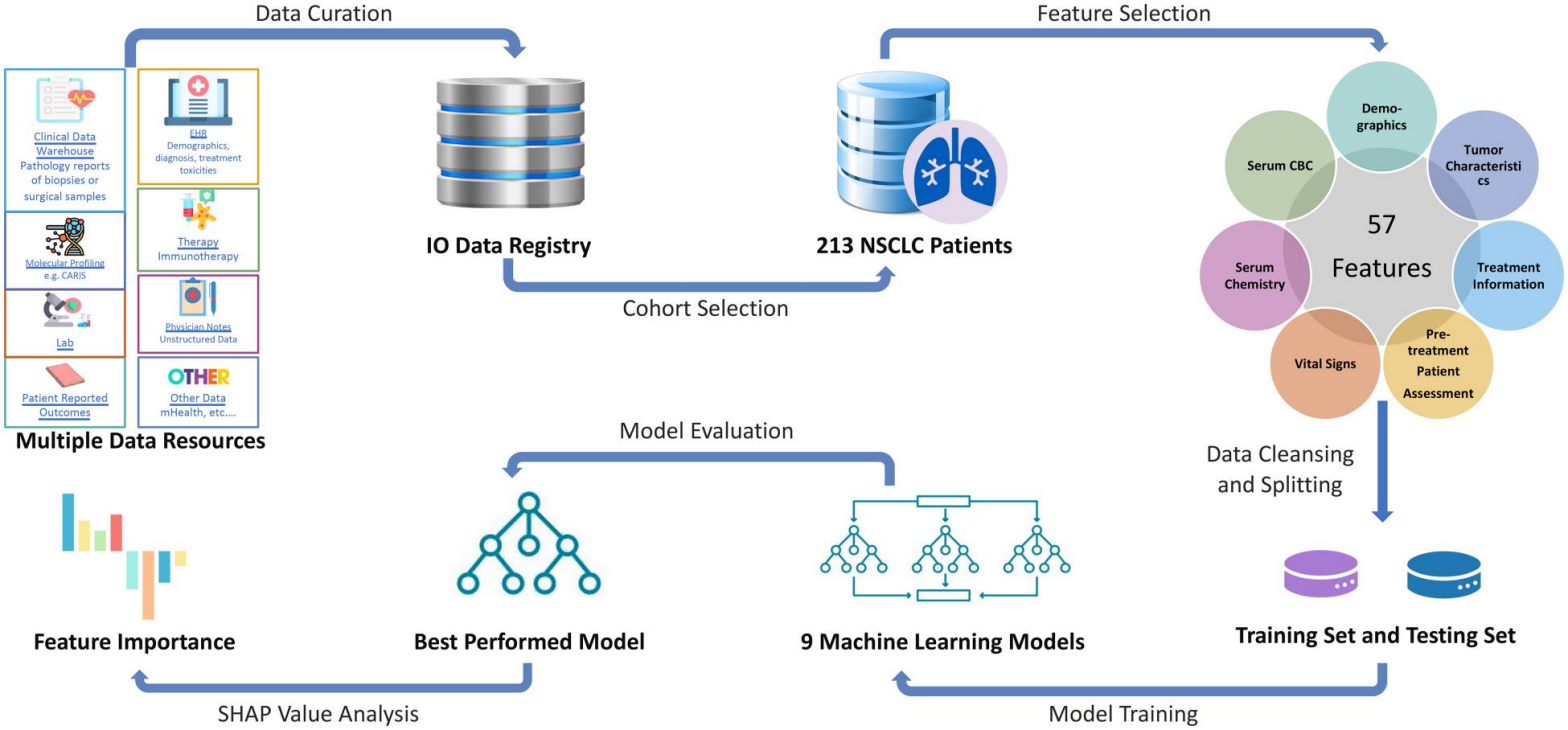
Study flowchart . This figure outlines the process from data curation and cohort selection to feature selection, model training and evaluation, and feature importance analysis. CBC: complete blood count; IO: Immuno-Oncology; SHAP: Shapley additive explanations.

A detailed flowchart of the methodology adopted is presented in Figure 1 for clarity and reference.

### Data source

The cohort investigated in this study was curated from the Immuno-Oncology (IO) database,^14^ a single-center repository developed by a multidisciplinary team of clinicians, bioinformatics engineers, and biostatisticians at the Georgetown-Lombardi Comprehensive Cancer Center. By leveraging technological advances and collaborating with partner hospitals, the repository was built with over 1400 patients from the 10 Washington DC and Baltimore MedStar Health network hospitals. Data sources include hospital medical records, laboratory data, pathology reports, radiology assessments, and cancer registry information. To facilitate the curation and entry of these data from patient charts, we employ REDCap^15^ as our electronic data capture tool. The repository has more than 100 features and includes 16 types of cancer and 11 kinds of ICI treatments. We identified patients with NSCLC who received ICI treatment with anti-PD-1 or anti-PD-L1 monoclonal antibodies between January 2011 and April 2018. Treatment responses for each patient were evaluated at 12 weeks after initiating immunotherapy using investigator assessed response based on RECIST 1.1. Patients who did not undergo treatment response assessment were excluded.

### Feature selection and data preprocessing

As the response variable, the treatment outcome is classified into a binary variable, categorizing patients as either responders or non-responders to the treatment. CR and PR are considered responding to the treatment, whereas PD and SD are classified as not responding to the treatment. For demographic features, we chose to include age at the onset of treatment, gender categorized as female or male, and race. We subsequently included 9 tumor-related features. These encompass the NSCLC histological subtype, stage, PD-L1 expression, mutation status of both Epidermal Growth Factor Receptor (EGFR) and KRAS genes, disease state (locally advance vs distant metastatic disease), and sites of metastasis. Additionally, we included 2 treatment-related attributes: the type of immunotherapy and the line of therapy. Three pretreatment characteristics were also identified for inclusion: pretreatment body mass index (BMI) level, baseline Eastern Cooperative Oncology Group (ECOG) performance status (PS), and smoking history. Comprehensive details on the pre-processing and classification methods applied to each feature can be found in the Supplementary material. Simultaneously, we included baseline laboratory tests and vital signs. We included only features with less than 30% missing data, to improve the reliability of our findings. Furthermore, we calculated the neutrophil-to-lymphocyte ratio (NLR), a prognostic biomarker of immune competence and recognized for its predictive value in immunotherapy outcomes, validated by previous studies.^16^^,^^17^ Missing data in categorical features were categorized as “Unknown” as a classification providing valuable clinical insights; further details regarding the “Unknown” categorization are available in the Supplementary material. Missing values in numerical features were imputed using the median value calculated from all samples of the respective features prior to training the machine learning models. The units and normal ranges for each laboratory test and vital sign are detailed in Table S1.

### Data description analysis

To gain a deeper understanding of the patient population and the factors influencing treatment response, we conducted a data descriptive analysis across 3 cohorts: the entire patient cohort, those who responded to treatment, and those who did not respond. For our analysis of numeric features, we calculated the mean and standard deviation within these 3 cohorts. Additionally, we employed Mann–Whitney U tests to assess the significance of differences in these numeric features between the cohort of responders and the cohort of non-responders. Regarding categorical features, we determined the frequency and percentage distribution of each category within the cohorts. We gauged the significance of disparities between the responder and non-responder cohorts by utilizing the Chi-squared test for categorical features. The significance was determined by a *P*-value at the level of .05.

### Model development, evaluation, and selection

We developed predictive machine learning models to evaluate NSCLC treatment response, following the normalization and standard scaling of numerical features, and converting each distinct category into a binary variable. To ensure robust model performance, models were trained with 80% of the data with 5-fold cross-validation and tested with the remaining 20% of the data. Nine distinct machine learning models were developed and trained to explore their effectiveness in predicting treatment response. These models encompassed a variety of techniques, including the following: Logistic Regression (LR), Support Vector Machine (SVM), Random Forest (RF), k-Nearest Neighbors (KNN), Bagging k-Nearest Neighbors (BKNN), Gradient Boosting (GB), Decision Tree Classifier (DTC), Bernoulli Naive Bayes (BNB), and eXtreme Gradient Boosting (XGB). To comprehensively assess the performance of these models, we adopted a range of evaluation metrics including precision, recall, accuracy, F1 score, and area under the receiver operating characteristic curve (AUROC).^18^ The AUROC for each model was plotted and compared with the AUROC of conventional prognostic PD-L1 as the baseline, and the best predictive model was selected by evaluating the AUROC score. This selected model will undergo further analysis, enabling us to unravel the pivotal factors influencing treatment response.

### Model interpretation

To gain a deeper insight into the predictive capabilities of the selected best model, we conducted an interpretation analysis using Shapley additive explanations (SHAP) values.^19^ Each SHAP value quantifies the impact of a feature on the model’s prediction, considering the interaction with other features. Positive SHAP values indicate a feature’s upward influence on model predictions, while negative values suggest a downward impact. The magnitude of a SHAP value reflects the strength of this influence. Aggregated SHAP values reveal the significance of features within the model, with larger absolute values signifying greater importance. Scatter plots of top features will be created to visually analyze how each one specifically affects the predictions.

## Results

### Patient characteristics

A total of 244 patients diagnosed with NSCLC were identified during the study period. We excluded 31 patients because their treatment response data was not available at the time of analysis. The final cohort included 213 patients, comprising 72 (34%) ICI responders and 141 (66%) ICI non-responders. Detailed patient characteristics categorized by treatment response are presented in Table 1, and a comprehensive summary of all available features is presented in Table S2. Laboratory tests and vital signs that showed significant differences between responder and non-responder groups, along with their normal ranges, are visualized in Figure S2. In the overall cohort, the mean age of patients age was 67.7 years with a standard deviation (SD) of 10.9, with 50.7% being female, 48.8% White, and 45.5% Black. Overall, similar distributions across the 2 response categories were observed. The overall cohort included 78.9% patients with NSCLC adenocarcinoma histology, 31.5% with PD-L1 score of 1+, 7% with +EGFR mutations, and 18.3% with +KRAS mutations. The most common metastatic sites were lung, bone, and lymph nodes. Overall, 30%, 55%, and 15% patients received ICI as first line (1L), 2L, and 2L+ treatment settings, respectively.

**Table 1.** Patient characteristics categorized by treatment response.

Characteristic, no. (%)	Entire cohort	Responders^a^	Non-responders
	N = 213	N = 72	N = 141
Age—years, mean (SD)	67.72 (10.90)	68.61 (10.80)	67.27 (10.93)
Gender—female	108 (50.70)	37 (51.39)	71 (50.35)
Race			
Black	97 (45.54)	32 (44.44)	65 (46.10)
White	104 (48.83)	33 (45.83)	71 (50.35)
Others	12 (5.63)	7 (9.72)	5 (3.55)
NSCLC type—adenocarcinoma	168 (78.87)	55 (76.39)	113 (80.14)
Stage—IV	153 (71.83)	57 (79.17)	96 (68.09)
Positive PD-L1 status	67 (31.46)	34 (47.22)	33 (23.40)
Positive EGFR	15 (7.04)	4 (5.56)	11 (7.80)
Positive KRAS	39 (18.31)	10 (13.89)	29 (20.57)
Metastatic disease present before Immuno-Oncology	195 (91.55)	63 (87.50)	132 (93.62)
Bone metastasis	71 (33.33)	18 (25.0)	53 (37.59)
Lung metastasis	96 (45.07)	29 (40.28)	67 (47.52)
Lymph nodes metastasis	130 (61.03)	42 (58.33)	88 (62.41)
Line of ICI treatment			
First	65 (30.52)	34 (47.22)	31 (21.99)
Second	117 (54.93)	37 (51.39)	80 (56.74)
Third or later	31 (14.55)	1 (1.39)	30 (21.28)
Pretreatment BMI, kg/m^2^			
BMI < 30	100 (46.95)	38 (52.78)	69 (48.94)
BMI ≥ 30	113 (53.05)	34 (47.22)	72 (51.06)
Pretreatment ECOG PS			
0-1	154 (72.30)	61 (84.72)	93 (65.96)
≥2	59 (27.70)	11 (15.28)	48 (34.04)
Smoking history			
Ever smokers^b^	170 (79.81)	62 (86.11)	108 (76.60)
Never smoker	43 (20.19)	10 (13.89)	33 (23.40)

BMI: body mass index; ECOG PS: Eastern Cooperative Oncology Group performance status; ICI: immune checkpoint inhibitor.

a Responders: include complete response (CR) or partial response (PR).

b Ever smokers: defined as current or ever had a history of smoking.

### Machine learning model and its performance

**Figure 2.**
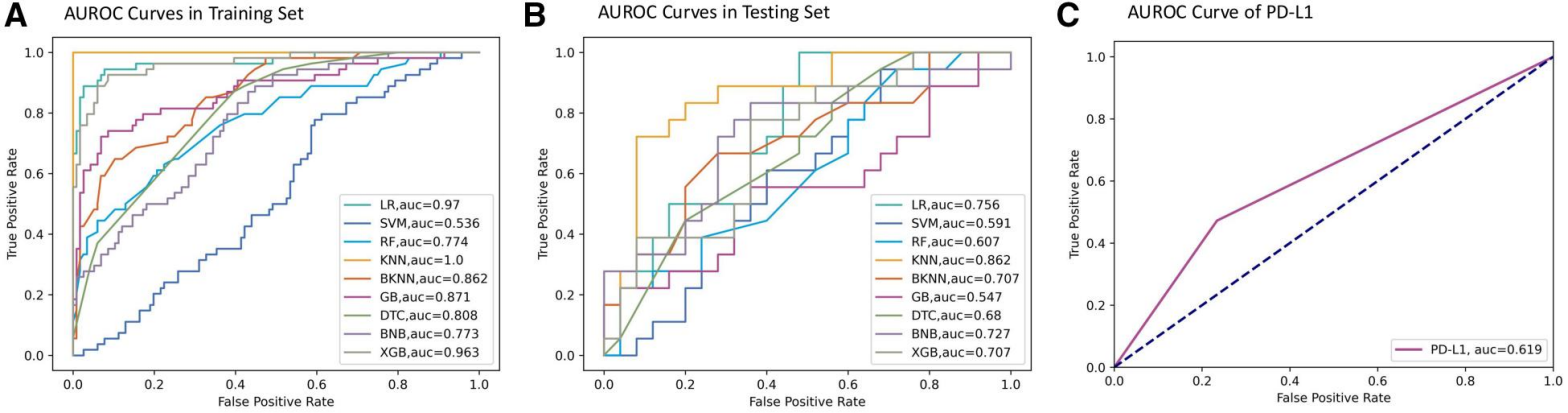
AUROC curves for (A) training set, (B) testing set, and (C) conventional prognostic biomarker PD-L1. The AUROC scores of LR, KNN, BKNN, DTC, BNB, and XGB on the testing set all outperform PD-L1. Among them, the KNN model achieved the highest AUROC of 0.862.

We identified 57 features to include in our machine learning model, including 3 demographic features, 9 tumor status features, 2 features represent treatment information, 3 features represent pre-treatment information, 15 serum complete blood counts (CBC) before treatment, 18 serum chemistry tests before treatment, 6 vital signs before treatment, and NLR (Table S2). Thirteen laboratory tests and vital signs show significant differences between the responders and non-responders, with comparative illustrations in Figure S1. A heat map showing the correlation between the features is presented in Figure S1. The division of the dataset by training on 80% and testing on 20% of the data yielded a training set comprising 170 patients and a testing set with 43 patients. The characteristics of the training and testing sets are displayed in Table S3. The performance metrics of the 9 machine learning models in both the training and testing sets are outlined in Table 2. Figure 2 presents the AUROC curves in the training set, testing set, and PD-L1. Based on the evaluation of the AUROC score in the testing set, it is evident that KNN outperforms the other models and PD-L1 with an AUROC score of 0.862.

**Table 2.** Performance scores for each machine learning model.

Model	Precision	Recall	Accuracy	F1	AUROC
LR	0.667	0.720	0.628	0.692	0.756
SVM	0.561	0.920	0.535	0.697	0.591
RF	0.581	1.000	0.581	0.735	0.607
KNN	0.581	1.000	0.581	0.735	0.862
BKNN	0.595	1.000	0.605	0.746	0.707
GB	0.629	0.880	0.628	0.733	0.640
DTC	0.667	0.800	0.651	0.727	0.680
BNB	0.641	1.000	0.674	0.781	0.727
XGB	0.656	0.840	0.651	0.737	0.707

BKNN: Bagging k-Nearest Neighbors; BNB: Bernoulli Naive Bayes; DTC: decision tree classifier; GB: gradient boosting; KNN: k-Nearest Neighbors; LR: logistic regression; RF: random forest; SVM: support vector machine; XGB: eXtreme Gradient Boosting (XGB).

### Model interpretation

**Figure 3.**
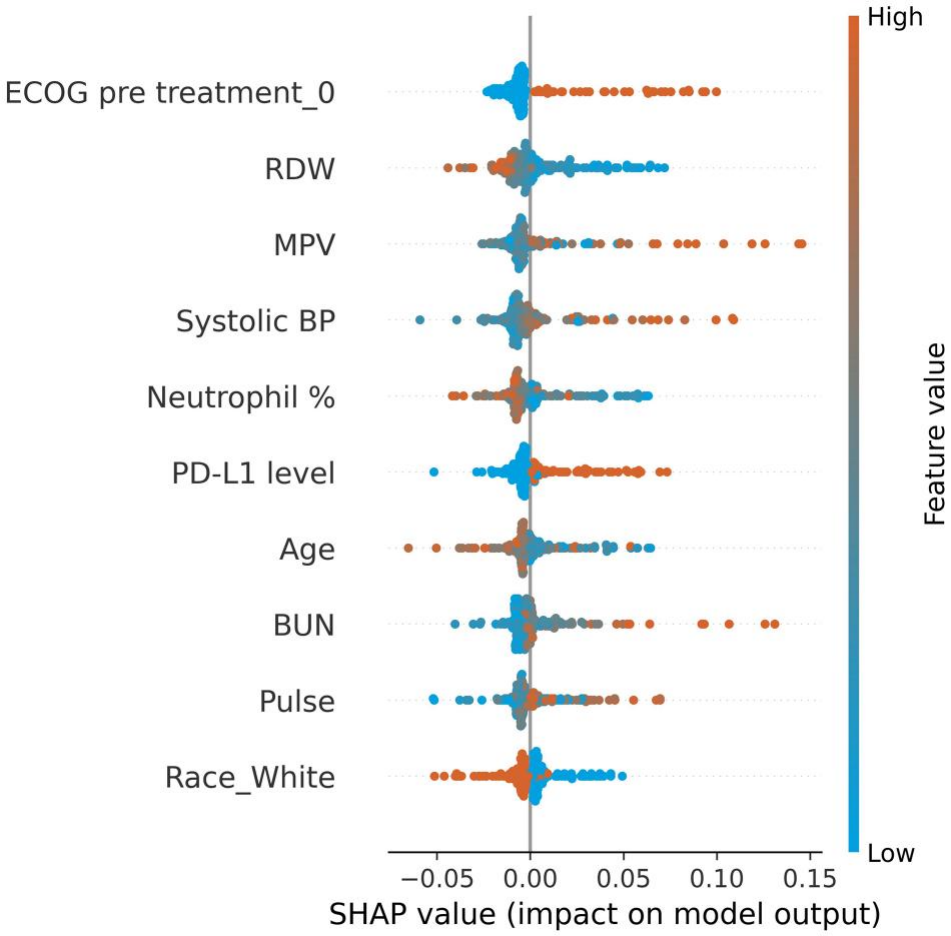
Top 10 features ranked by SHAP value. The top 10 ranked features are: ECOG performance status of 0 prior to treatment, red cell distribution width (RDW), mean platelet volume (MPV), systolic blood pressure (BP), neutrophil percentage, PD-L1 expression level, age, blood urea nitrogen (BUN), pulse rate, and White race. Features are ranked from top to bottom by their impact. Each dot represents a feature’s impact on the model’s output for an individual patient. Red dots indicate higher feature values and blue dots represent lower values. The position on the x-axis shows the SHAP value, with more positive values indicating a higher likelihood of immunotherapy response. Features are ranked from top to bottom by their impact. Rankings of SHAP value for all features can be found in Figure S3. The bottom 10 features ranked by SHAP value can be found in Figure S4.

Given the inherent randomization within the KNN model, we computed the mean of SHAP values across 20 results to enhance robustness. The SHAP values of each feature are shown in Figure S3, which illustrates the distribution and directionality of each feature’s influence on the predictive model, with the top 10 features shown in Figure 3 and the bottom 10 features shown in Figure S4. Top-ranked features such as ECOG PS of 0, red blood cell distribution width (RDW), mean platelet volume (MPV), systolic blood pressure (SBP), percentage of neutrophils, and PD-L1 level significantly influenced the model’s predictions. Specifically, ECOG PS of 0, a positive PD-L1 expression level, higher MPV values, and higher SBP correlated with increased treatment effectiveness. Conversely, lower RDW levels, and lower neutrophil percentages were also predictors of treatment response. Features at the bottom of the plot, such as underweight pretreatment BMI, EGFR status, pre-existing metastatic disease, and immunotherapy with anti-PD-1 plus chemotherapy, showed minimal impact on the model’s predictions, as evidenced by their SHAP values nearing zero. The rankings of feature importance in the model, both overall and within specific groups as determined by SHAP values, are detailed in Table S4. Scatter plots of SHAP values are shown in Figure S5, followed by a detailed description.

## Discussion

We conducted a comprehensive, multimodal, retrospective study to evaluate the role of physiological, psychosocial, and tumor microenvironmental risk factors in the efficacy of ICIs for NSCLC patients. Utilizing machine learning models, we assessed the influence of various predictive biomarkers related to ICI treatment. Our findings indicate the significant impact of a patient’s functional status, psychosocial risk factors such as SBP, inflammatory serum biomarkers, and tumor microenvironmental factors, including the expression of PD-1, on ICI treatment efficacy.

KNN’s outperformance than other models such as XGBoost may be due to the relatively small sample size, low feature dimensionality, and simplified binary encoding of most categorical variables, which can favor instance-based algorithms like KNN. Additionally, the strong signal in certain features may have allowed KNN’s distance-based approach to effectively distinguish responders from non-responders without the need for complex model structures.

The model identified an ECOG PS of 0 as a key predictor of ICI treatment response. Among those who responded, 33.3% had an ECOG PS of 0, compared to only 9.2% of non-responders (*P* = 0), supporting literature that associates better outcomes for patients with higher ECOG PS scores. Overall, an ECOG PS of 0 indicates good physiological health, as these patients are fully active and can perform all usual daily activities without restriction.^20^^,^^21^ This suggests they possess robust physical health, which may contribute to a more favorable immune response to immunotherapy. Interestingly, we also observed that higher SBP was associated with a better response to ICI treatment. The average SBP among responders was 134.03 ± 21.75 mm Hg versus 124.24 ± 17.08 mm Hg in non-responders (*P* = 0). SBP is vital in assessing allostatic load (AL),^22^ a cumulative measure of physiological stress resulting from chronic environmental stressors. AL is typically derived from measurements across 4 physiological systems: cardiovascular (heart rate, SBP, and diastolic blood pressure), metabolic (BMI, alkaline phosphatase, blood glucose, and albumin), renal (creatinine and blood urea nitrogen), and immune (white blood cell count). Traditionally, AL has been associated with poor cancer outcomes.^23^ However, the findings from our study are intriguing, as chronic stress is usually correlated with worse cancer prognosis. It is important to note that most studies in this area have focused on chemotherapy treatments, and the impact of AL on the efficacy of ICI treatment warrants further investigation.

The model revealed that lower RDW, MPV, and a lower percentage of neutrophils at baseline can predict a positive response to ICIs. RDW, which measures the variability in the size of red blood cells, has been linked to inflammation^24^ and poor prognosis in various conditions, including anemia^25^ and sarcopenia.^26^ Furthermore, elevated RDWs are associated with worse clinical outcomes in patients with lung cancer.^27–29^ MPV is also related to inflammation and the production of platelets and serves as a marker for platelet activation.^30^^,^^31^ A higher percentage of neutrophils at baseline has been correlated with impaired T-cell immunity and poorer cancer outcomes.^32^^,^^33^ These laboratory findings suggest that chronic systemic inflammation may impact the effectiveness of ICIs. This long-term immunosuppressive state is likely triggered by the stimulation of M2 macrophages by cancer cells, which release cytokines such as TGF-β, IL-6, and IL-10, all of which can weaken the immune response.^34^ This underlying inflammation can alter the tumor microenvironment, modify immune cell behavior, influence cytokine profiles, and affect the expression of immune checkpoint proteins—factors that are crucial for the success of immunotherapy. Therefore, our study emphasizes the importance of addressing underlying inflammation before initiating treatment as a novel strategy to enhance the efficacy of immunotherapeutic approaches.

PD-L1 expression is another significant factor, per our predictive model. Furthermore, 47.22% of responders had PD-L1 levels exceeding 5%, which is considered intermediate or high; in contrast, only 23.4% of non-responders met the same criteria (*P* = 0). This emphasizes the importance of PD-L1 as a biomarker for selecting patients who are more likely to benefit from immunotherapies. PD-L1 as a protein expressed in tumor cells, interacts with PD-1 on T-cells to inhibit immune responses, allowing cancer cells to evade immune detection.^35^ Thus, therapies that target the PD-1/PD-L1 axis can reactivate the immune system against the tumor, reducing the tumor’s immunosuppressive properties and enabling the immune system to fight cancer more effectively. Our findings reinforce the value of utilizing PD-L1 expression as a tool in guiding treatment decisions for NSCLC patients.^36^ However, although PD-L1 and EGFR are widely recognized biomarkers for ICI response in NSCLC, they did not emerge as top features in the prediction model. This may be attributed to the skewed distribution in the cohort, where nearly half of the patients lacked PD-L1 testing, and only 7.04% were EGFR positive, which potentially dilutes their predictive strength. Moreover, as shown in Figure 2, PD-L1 alone demonstrates limited predictive power for tumor response in the full patient cohort. To further evaluate this, we analyzed the AUROC of PD-L1 and EGFR in both the full cohort and the subgroups of patients who underwent PD-L1 or EGFR testing, as presented in Figure S6. These additional analyses consistently demonstrate the limited predictive performance of PD-L1 and EGFR, even among tested patients. Therefore, beyond the substantial proportion of unteseted patients, the limited predictive power observed for PD-L1 and EGFR is likely another factor contributing to their relatively lower importance rankings in our model.

This study presents an exciting opportunity to enhance our understanding, though it does have some limitations. The specific scope and integrity of the data source led to a smaller and single-institution dataset for training our machine learning model. However, our model outperformed traditional prognostic biomarkers, such as PD-L1, demonstrating its potential to surpass current clinical practices in predictive accuracy. Despite that, the data we collected and analyzed represent a comprehensive integration of phenotypic and genotypic features, which is uncommon in current literature. Although our patient cohort is relatively small, the curation and development of such a dataset is resource-intensive. This opens the door for future research to expand our dataset and explore new avenues for improvement. Moreover, ICI-treated cohort in this study limits distinguishing predictive features specific to ICI sensitivity from general prognosis indicators. Future studies comparing ICI and non-ICI cohorts will validate the specificity of these prognostic features for immunotherapy response. Additionally, the bottom 10 features ranked by SHAP values suggest areas for further investigation, highlighting the potential for deeper insights. Our model’s high AUROC score demonstrates robust predictive capabilities, reinforcing the benefits of integrating a broad spectrum of phenotypic and genotypic features into machine learning algorithms for significant predictive accuracy. Nevertheless, extrapolating the most and least influential features identified by our model to clinical practice requires rigorous validation. While these findings suggest promising directions for future research, they should not be seen as definitive until further confirmed in comprehensive clinical trials and real-world studies. The novelty of our results lies in their potential to guide subsequent investigations to refine NSCLC patient care. In the future, collaborations will be pursued to collect multi-institutional patient data, aiming to expand the training set and enable external validation of the model.

## Conclusion

This study utilized a comprehensive dataset of phenotypic and genotypic information to conduct an in-depth analysis of treatment responses in patients with NSCLC receiving ICI treatments. The machine learning model exhibited a strong predictive capability for treatment outcomes, surpassing that of the traditionally used clinical prognostic marker PD-L1. Our findings highlight the complex interactions among various risk factors that influence the effectiveness of ICI. Further prospective studies on multimodal biomarkers are needed, particularly those that integrate patients’ functional status, psychosocial risk factors, and elements that affect the tumor microenvironment.

## Supplementary Material

ooaf069_Supplementary_Data

## Data Availability

The data underlying this article cannot be shared publicly to protect patient privacy. The data will be shared on reasonable request to the corresponding author.
