# Supplementary material for: Utilizing Immuno-Oncology registry data for enhanced non-small cell lung cancer treatment predictions
Source: JAMIA Open. 2025 Jul 9;8(4):ooaf069. doi: 10.1093/jamiaopen/ooaf069 (PMC12239864; doi:10.1093/jamiaopen/ooaf069)
Supplement: ooaf069_Supplementary_Data [file ooaf069_supplementary_data.docx]

**Supplemental Material**

**Preprocessing and Classification Applied to Each Feature**

# Definition of NSCLC Characteristics

# The subtypes of NSCLC were classified as adenocarcinoma, with squamous cell and large cell carcinomas grouped due to their less frequent occurrence in the dataset. Given that the majority of the patients were diagnosed with stage IV cancer, which includes sub-stages IVA and IVB, we categorized cancer stages into two groups: “Stage IV” and “Other Stages”. The “Other Stages” group comprises stages IB, II, IIA, IIB, IIIA, IIIC, and IIIB, as well as cases with incomplete records. Patients’ PD-L1 levels were categorized as “Positive” if they were 5% or higher50. Those with levels below 5%, as well as those not tested, were grouped under “Negative/Unknown”. The status of EGFR and KRAS mutations was categorized as “Mutation Present” for detected mutations. Cases with no detected mutations, known as wild type, along with those of unknown mutation status, were collectively classified as “Wild Type/Unknown”. Other mutations, including ALK, BRAF, and ROS, were excluded from the analysis due to their relatively rare incidence in NSCLC cases. Similarly, metastases to the brain, liver, peritoneum, and other less common sites were not included in our analysis due to their infrequent presence in our NSCLC cohort.

# Definition of Immune Checkpoint Inhibitors (ICIs)

# The current immunotherapies were categorized as Anti PD-1 therapies (such as Nivolumab and Pembrolizumab), Anti PD-L1 therapies (including Atezolizumab, Durvalumab, and Avelumab), and combinations of Anti PD-1 with chemotherapy (specifically Carboplatin plus Pembrolizumab plus Pemetrexed). Therapy lines were designated as first, second, or third and above, with the latter group combining lines three and four due to their smaller numbers in our cohort.

# Definition of Other Pretreatment Patient Characteristics

# Race was classified into White, Black, or African American, and an “Other/Unknown” category, which encompasses Asian, races not specified, and unknown races, due to their smaller representation in the dataset. Pretreatment BMI levels were categorized as normal, overweight, and underweight, using thresholds of 18.5 and 30. ECOG performance status prior to treatment was categorized into scores of 0, 1, and 2 or unknown, including unknown scores. Smoking history was delineated as current smoker, former smoker, and never smoker, with the latter group combined with unknown status under “Never Smoker/Unknown”. Correlations between all chosen features for model training were examined to ensure minimal redundancy.

**Supplemental Table 1. Units and Normal Range for Laboratory Tests and Vital Signs**

| ***Laboratory Test*** | ***Normal Range*** |
| --- | --- |
| ***Serum CBC before Treatment*** | |
| Basophils | 0.5 - 1.0% |
| Eosinophils | 0.5 – 4% |
| Hematocrit | 38.3 - 48.6% |
| Hemoglobin | 13.2 - 17.1 g/dL |
| Lymphocytes | 1.0 - 4.0 x 10^9^/L |
| Mean Corpuscular Hemoglobin (MCH) | 27 - 31 pg/cell |
| Mean Corpuscular Hemoglobin Concentration (MCHC) | 32 - 36 g/dL |
| Mean Corpuscular Volume (MCV) | 80 - 100 fL |
| Mean Platelet Volume (MPV) | 7.4 - 10.4 fL |
| Monocytes | 0.2 - 0.8 x 10^9^/L |
| Neutrophil % | 40 - 60% |
| NLR | Normal range varies by laboratory |
| Platelet | 150 - 450 x 10^3^/mm³ |
| Red Blood Cell (RBC) Count | 4.5 - 5.5 million/mm³ |
| Red Cell Distribution Width (RDW) | 11.5 - 14.5% |
| White Blood Cell (WBC) Count | 4 - 11 x 10^3^/mm³ |
| ***Serum Chemistry before Treatment*** | |
| Albumin to Globulin (A/G) Ratio | 1.2 - 2.2 |
| Alanine Aminotransferase (ALT) | 7 - 56 IU/L |
| Aspartate Aminotransferase (AST) | 5 - 40 IU/L |
| Albumin | 3.4 - 5.4 g/dL |
| Alkaline Phosphatase | 44 - 147 IU/L |
| Anion Gap | 8 - 16 mmol/L |
| Bilirubin | 0.1 - 1.2 mg/dL |
| Blood Urea Nitrogen (BUN) | 7 - 20 mg/dL |
| Calcium | 8.5 - 10.5 mg/dL |
| Carbon Dioxide | 23 - 29 mmol/L |
| Chloride | 96 - 106 mmol/L |
| Creatinine | 0.84 - 1.21 mg/dL |
| Creatinine Clearance | 100 - 150 mg/dL |
| Globulin | 2.0 - 3.9 g/dL |
| Glucose | 70 - 99 mg/dL |
| Potassium | 3.5 - 5.3 mmol/L |
| Protein | 6.0 - 8.3 g/dL |
| Sodium | 135 - 145 mmol/L |
| ***Vital Signs before Treatment*** | |
| Body Surface Area (BSA) | - |
| Diastolic | 60 - 80 mm Hg |
| Systolic | 90 - 120 mm Hg |
| Pulse | 60 - 100 beats per minute |
| Pulse Oximetry | 95 - 100% |
| Respiration | 12 - 20 breaths per minute |

**Supplemental Table 2: Detailed Description of Patient Information by Feature.** Numeric features are presented as mean ± standard deviation and categorical features are described with their corresponding percentages (in parentheses).

| **Characteristic, no (%)** | **Entire Cohort**  **N=213** | **Positive Responders**  **N=72** | **Negative Responders**  **N=141** | ***p-value*** | |
| --- | --- | --- | --- | --- | --- |
| ***Demographics*** | | | | |  |
| **Age—years, mean (SD)** | 67.72 (±10.9) | 68.61 (±10.8) | 67.27 (±10.93) | 0.62 | |
| **Gender** |  |  |  | 1 | |
| Female | 108 (50.7) | 37 (51.39) | 71 (50.35) |  | |
| Male | 105 (49.3) | 35 (48.61) | 70 (49.65) |  | |
| **Race** |  |  |  | 0.18 | |
| Black | 97 (45.54) | 32 (44.44) | 65 (46.10) |  | |
| White | 104 (48.83) | 33 (45.83) | 71 (50.35) |  | |
| Other/Unknown | 12 (5.63) | 7 (9.72) | 5 (3.55) |  | |
| ***Tumor Status*** | | | | |  |
| **NSCLC Type—adenocarcinoma** | 168 (78.87) | 55 (76.39) | 113 (80.14) | 0.12 | |
| **Stage—IV** | 153 (71.83) | 57 (79.17) | 96 (68.09) | 0.65 | |
| **PD-L1 Status** |  |  |  | **0** | |
| Positive | 67 (31.46) | 34 (47.22) | 33 (23.4) |  | |
| Negative/Unknown | 146 (68.54) | 38 (52.78) | 108 (76.6) |  | |
| **EGFR Mutation Status** |  |  |  | 0.75 | |
| Mutation Present | 15 (7.04) | 4 (5.56) | 11 (7.8) |  | |
| Wild Type/Unknown | 198 (92.96) | 68 (94.44) | 130 (92.2) |  | |
| **KRAS Mutation Status** |  |  |  | 0.31 | |
| Mutation Present | 39 (18.31) | 10 (13.89) | 29 (20.57) |  | |
| Wild Type/Unknown | 174 (81.69) | 62 (86.11) | 112 (79.43) |  | |
| **Metastatic Disease Present before IO** | 195 (91.55) | 63 (87.50) | 132 (93.62) | 0.21 | |
| **Bone metastasis** | 71 (33.33) | 18 (25.0) | 53 (37.59) | 0.09 | |
| **Lung metastasis** | 96 (45.07) | 29 (40.28) | 67 (47.52) | 0.39 | |
| **Lymph nodes metastasis** | 130 (61.03) | 42 (58.33) | 88 (62.41) | 0.67 | |
| ***Treatment Information*** | | | | |  |
| **Current ICI therapy** |  |  |  | 0.06 | |
| Anti PD-1 monotherapy | 166 (77.93) | 50 (69.44) | 116 (82.27) |  | |
| Anti PD-L1 monotherapy | 27 (12.68) | 11 (15.28) | 16 (11.35) |  | |
| Anti PD-1 + chemotherapy | 20 (9.39) | 11 (15.28) | 9 (6.38) |  | |
| **Line of ICI therapy** |  |  |  | **0** | |
| 1 | 65 (30.52) | 34 (47.22) | 31 (21.99) |  | |
| 2 | 117 (54.93) | 37 (51.39) | 80 (56.74) |  | |
| ≥3 | 31 (14.55) | 1 (1.39) | 30 (21.28) |  | |
| ***Pre-Treatment Information*** | | | | |  |
| **BMI** |  |  |  | 0.41 | |
| Normal | 100 (46.95) | 38 (52.78) | 69 (48.94) |  | |
| Overweight | 100 (46.95) | 31 (43.06) | 62 (43.97) |  | |
| Underweight | 13 (6.10) | 3 (4.17) | 10 (7.09) |  | |
| **ECOG PS** |  |  |  | **0** | |
| 0 | 37 (17.37) | 24 (33.33) | 13 (9.22) |  | |
| 1 | 117 (54.93) | 37 (51.39) | 80 (56.74) |  | |
| ≥2 or Unknown | 59 (27.70) | 11 (15.28) | 48 (34.04) |  | |
| **Smoking history** |  |  |  | 0.1 | |
| Current Smoker | 32 (15.02) | 15 (20.83) | 17 (12.06) |  | |
| Previous Smoker | 138 (64.79) | 47 (65.28) | 91 (64.54) |  | |
| Never Smoker/Unknown | 43 (20.19) | 10 (13.89) | 33 (23.40) |  | |
| ***Serum CBC before Treatment*** | | | | |  |
| **Basophils** | 0.01 (±0.03) | 0.01 (±0.03) | 0.01 (±0.03) | 0.99 | |
| **Eosinophils** | 0.15 (±0.30) | 0.17 (±0.18) | 0.14 (±0.34) | **0.02** | |
| **Hematocrit** | 34.81 (±6.08) | 36.69 (±5.67) | 33.88 (±6.07) | **0** | |
| **Hemoglobin** | 11.26 (±2.13) | 11.91 (±1.98) | 10.93 (±2.13) | **0** | |
| **Lymphocytes** | 1.33 (±0.73) | 1.45 (±0.79) | 1.27 (±0.69) | 0.1 | |
| **MCH** | 29.05 (±3.25) | 29.29 (±3.49) | 28.93 (±3.11) | 0.38 | |
| **MCHC** | 32.28 (±1.38) | 32.41 (±1.33) | 32.21 (±1.39) | 0.29 | |
| **MCV** | 89.92 (±8.37) | 90.23 (±9.11) | 89.76 (±7.97) | 0.69 | |
| **MPV** | 9.47 (±0.89) | 9.65 (±0.99) | 9.38 (±0.83) | 0.12 | |
| **Monocytes** | 0.67 (±0.36) | 0.62 (±0.28) | 0.70 (±0.40) | 0.4 | |
| **Neutrophil %** | 68.71 (±12.72) | 66.39 (±12.16) | 69.87 (±12.84) | **0.05** | |
| **NLR** | 5.58 (±5.11) | 4.33 (±3.06) | 6.21 (±5.78) | **0.02** | |
| **Platelet** | 271.88 (±120.31) | 257.45 (±87.34) | 279.10 (±133.20) | 0.69 | |
| **RBC** | 3.89 (±0.70) | 4.10 (±0.68) | 3.79 (±0.70) | **0.01** | |
| **RDW** | 16.06 (±2.61) | 15.06 (±1.89) | 16.56 (±2.78) | **0** | |
| **WBC** | 7.97 (±5.01) | 7.48 (±3.70) | 8.21 (±5.53) | 0.6 | |
| ***Serum Chemistry before Treatment*** | | | | |  |
| **A/G Ratio** | 0.98 (±0.32) | 1.07 (±0.3) | 0.94 (±0.31) | **0** | |
| **ALT** | 29.28 (±66.97) | 35.04 (±107.15) | 26.4 (±31.03) | 0.61 | |
| **AST** | 23.46 (±19.57) | 21.28 (±22.02) | 24.55 (±18.12) | **0.01** | |
| **Albumin** | 3.39 (±0.56) | 3.60 (±0.53) | 3.29 (±0.54) | **0** | |
| **Alkaline Phosphatase** | 104.27 (±47.73) | 97.15 (±27.86) | 107.84 (±54.7) | 0.76 | |
| **Anion Gap** | 7.91 (±2.33) | 7.57 (±2.09) | 8.07 (±2.41) | 0.15 | |
| **Bilirubin** | 0.43 (±0.26) | 0.41 (±0.20) | 0.44 (±0.29) | 0.61 | |
| **BUN** | 16.59 (±7.58) | 18.01 (±8.12) | 15.87 (±7.19) | 0.06 | |
| **Calcium** | 9.03 (±0.60) | 9.18 (±0.54) | 8.96 (±0.62) | **0.02** | |
| **Carbon Dioxide** | 26.27 (±3.41) | 26.03 (±3.26) | 26.40 (±3.47) | 0.68 | |
| **Chloride** | 102.14 (±4.39) | 102.63 (±4.24) | 101.90 (±4.45) | 0.3 | |
| **Creatinine** | 0.92 (±0.34) | 0.95 (±0.34) | 0.91 (±0.34) | 0.44 | |
| **Creatinine Clearance** | 81.71 (±37.71) | 80.41 (±37.74) | 82.34 (±37.68) | 0.85 | |
| **Globulin** | 3.66 (±0.71) | 3.57 (±0.69) | 3.70 (±0.72) | 0.12 | |
| **Glucose** | 115.94 (±43.48) | 124.84 (±55.53) | 111.49 (±35.13) | 0.21 | |
| **Potassium** | 4.27 (±0.45) | 4.39 (±0.45) | 4.21 (±0.43) | **0.02** | |
| **Protein** | 7.05 (±0.65) | 7.18 (±0.51) | 6.99 (±0.70) | 0.07 | |
| **Sodium** | 138.15 (±3.73) | 138.51 (±3.72) | 137.98 (±3.72) | 0.2 | |
| ***Vital Signs before Treatment*** | | | | |  |
| **BSA** | 1.82 (±0.24) | 1.83 (±0.22) | 1.82 (±0.26) | 0.49 | |
| **Diastolic Blood Pressure** | 72.73 (±10.6) | 74.54 (±10.61) | 71.78 (±10.47) | 0.09 | |
| **Systolic Blood Pressure** | 127.58 (±19.37) | 134.03 (±21.75) | 124.24 (±17.08) | **0** | |
| **Pulse** | 89.02 (±16.08) | 86.06 (±15.97) | 90.56 (±15.92) | 0.07 | |
| **Pulse Oximetry** | 96.84 (±2.38) | 96.83 (±2.31) | 96.84 (±2.42) | 0.9 | |
| **Respiration** | 18.69 (±4.63) | 19.35 (±7.31) | 18.35 (±2.13) | 0.31 | |

**Supplemental Table 3. Patient Characteristics in Training and Testing Sets**. Age is represented by mean ± standard deviation, while percentages for other features are provided in parentheses.

|  | **Training Set**  **N=170** | **Testing Set**  **N=43** |
| --- | --- | --- |
| **Treatment response** |  |  |
| Responder | 54 (31.76) | 18 (41.86) |
| Non-Responder | 116 (68.24) | 25 (58.14) |
| ***Demographics*** | | |
| **Age—years, mean (SD)** | 68.01 (±10.61) | 66.58 (±11.93) |
| **Gender** |  |  |
| Female | 85 (50.0) | 23 (53.49) |
| Male | 85 (50.0) | 20 (46.51) |
| **Race** |  |  |
| Black | 78(45.88) | 19(44.19) |
| White | 82(48.24) | 22(51.16) |
| Other/Unknown | 10(5.88) | 2(4.65) |
| ***Tumor Characteristics*** | | |
| **NSCLC Type—adenocarcinoma** | 122 (71.76) | 31 (72.09) |
| **Stage—IV** | 135 (79.41) | 33 (76.74) |
| **PD-L1 Status** |  |  |
| Positive | 52 (30.59) | 15 (34.88) |
| Negative/Unknown | 118 (69.41) | 28 (65.12) |
| **EGFR Mutation Status** |  |  |
| Mutation Present | 13 (7.65) | 2 (4.65) |
| Wild Type/Unknown | 157 (92.35) | 41 (95.35) |
| **KRAS Mutation Status** |  |  |
| Mutation Present | 31 (18.24) | 8 (18.6) |
| Wild Type/Unknown | 139 (81.76) | 35 (81.4) |
| **Metastatic Disease** | 157 (92.35) | 38 (88.37) |
| **Bone Metastasis** | 56 (32.94) | 15 (34.88) |
| **Lung Metastasis** | 77 (45.29) | 19 (44.19) |
| **Lymph Nodes Metastasis** | 102 (60.0) | 28 (65.12) |
| ***Treatment Information*** | | |
| **Current ICI therapy** |  |  |
| Anti PD-1 monotherapy | 130 (76.47) | 36 (83.72) |
| Anti PD-L1 monotherapy | 24 (14.12) | 4 (9.30) |
| Anti PD-1 + chemotherapy | 16 (9.41) | 3 (6.98) |
| **Line of immunotherapy** |  |  |
| 1 | 48 (28.24) | 17 (39.53) |
| 2 | 97 (57.06) | 20 (46.51) |
| ≥3 | 25 (14.71) | 6 (13.95) |
| ***Pretreatment Patient Assessment*** | | |
| **BMI** |  |  |
| Normal | 80 (47.06) | 20 (46.51) |
| Overweight | 80 (47.06) | 20 (46.51) |
| Underweight | 10 (5.88) | 3 (6.98) |
| **ECOG PS** |  |  |
| 0 | 31 (18.24) | 6 (13.95) |
| 1 | 88 (51.76) | 29 (67.44) |
| ≥2 or Unknown | 51 (30.00) | 8 (18.60) |
| **Smoking history** |  |  |
| Current Smoker | 26 (15.29) | 6 (13.95) |
| Previously Smoker | 109 (64.12) | 29 (67.44) |
| Never Smoker/Unknown | 35 (20.59) | 8 (18.60) |

**Supplemental Table 4. SHAP value ranking for Each Feature**

|  | **SHAP Ranking Overall** | **SHAP Ranking in Group** |
| --- | --- | --- |
| ***Demographics*** |  |  |
| **Age** | 7 | 1 |
| **Gender** | 46 | 3 |
| **Race** |  |  |
| Black | 51 | 4 |
| White | 10 | 2 |
| Other/Unknown | 65 | 5 |
| ***Tumor Characteristics*** |  |  |
| **NSCLC Type—adenocarcinoma** | 57 | 5 |
| **Stage—IV** | 62 | 7 |
| **PD-L1 Status** | 6 | 1 |
| **EGFR Mutation Status** | 68 | 9 |
| **KRAS Mutation Status** | 61 | 6 |
| **Metastatic Disease Present before IO** | 67 | 8 |
| **Bone Metastasis** | 56 | 4 |
| **Lung Metastasis** | 25 | 2 |
| **Lymph nodes Metastasis** | 48 | 3 |
| ***Treatment Information*** |  |  |
| **Current ICI therapy** |  |  |
| Anti PD-1 monotherapy | 59 | 3 |
| Anti PD-L1 monotherapy | 64 | 5 |
| Anti PD-1 + chemotherapy | 66 | 6 |
| **Line of immunotherapy** |  |  |
| 1 | 35 | 1 |
| 2 | 52 | 2 |
| ≥3 | 60 | 4 |
| ***Pretreatment Patient Assessment*** |  |  |
| **BMI** |  |  |
| Normal | 31 | 4 |
| Overweight | 23 | 2 |
| Underweight | 69 | 9 |
| **ECOG PS** |  |  |
| 0 | 1 | 1 |
| 1 | 26 | 3 |
| ≥2 or Unknown | 49 | 6 |
| **Smoking history** |  |  |
| Current Smoker | 58 | 8 |
| Previously Smoker | 45 | 5 |
| Never Smoker/Unknown | 54 | 7 |
| ***Serum CBC before Treatment*** |  |  |
| **Basophils** | 55 | 16 |
| **Eosinophils** | 50 | 15 |
| **Hematocrit** | 28 | 7 |
| **Hemoglobin** | 19 | 5 |
| **Lymphocytes** | 17 | 4 |
| **MCH** | 34 | 9 |
| **MCHC** | 21 | 6 |
| **MCV** | 37 | 10 |
| **MPV** | 3 | 2 |
| **Monocytes** | 44 | 13 |
| **Neutrophil %** | 5 | 3 |
| **NLR** | 42 | 11 |
| **Platelet** | 47 | 14 |
| **RBC** | 32 | 8 |
| **RDW** | 2 | 1 |
| **WBC** | 43 | 12 |
| ***Serum Chemistry before Treatment*** |  |  |
| **A/G Ratio** | 24 | 9 |
| **ALT** | 63 | 18 |
| **AST** | 30 | 11 |
| **Albumin** | 18 | 7 |
| **Alkaline Phosphatase** | 38 | 14 |
| **Anion Gap** | 16 | 6 |
| **Bilirubin** | 53 | 17 |
| **BUN** | 8 | 1 |
| **Calcium** | 29 | 10 |
| **Carbon Dioxide** | 33 | 12 |
| **Chloride** | 15 | 5 |
| **Creatinine** | 13 | 3 |
| **Creatinine Clearance** | 14 | 4 |
| **Globulin** | 40 | 15 |
| **Glucose** | 11 | 2 |
| **Potassium** | 20 | 8 |
| **Protein** | 36 | 13 |
| **Sodium** | 41 | 16 |
| ***Vital Signs before Treatment*** |  |  |
| **BSA** | 12 | 3 |
| **Diastolic Blood Pressure** | 27 | 5 |
| **Systolic Blood Pressure** | 4 | 1 |
| **Pulse** | 9 | 2 |
| **Pulse Oximetry** | 39 | 6 |
| **Respiration** | 22 | 4 |


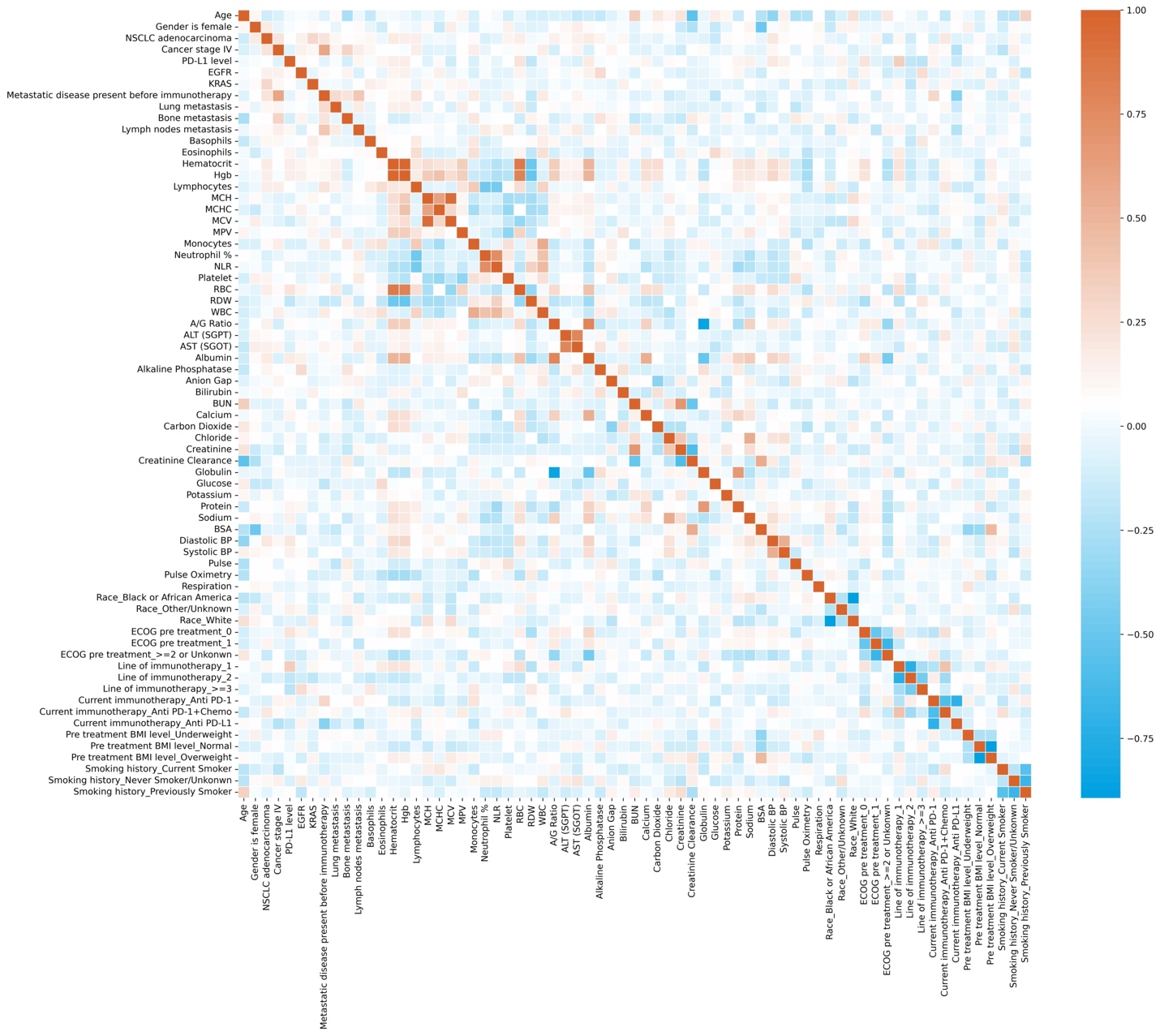
 **Supplemental Figure 1.** **Correlation Heatmap of Clinical Features.** This heatmap visualizes the pairwise correlation coefficients between clinical features used in the machine learning model for NSCLC treatment response prediction. Each cell represents the correlation between two features: red indicates a positive correlation, blue indicates a negative correlation, and the intensity of the color denotes the strength of the relationship. A value of 1 represents a perfect positive correlation, -1 represents a perfect negative correlation, and 0 indicates no correlation. Diagonal cells, which are the darkest red, represent the correlation of each feature with itself (perfect correlation). Hgb, MCH, MCHC, MCV, and ALT stand for hemoglobin, mean corpuscular hemoglobin, mean corpuscular hemoglobin concentration, mean corpuscular volume, and alanine aminotransferase, respectively.

#
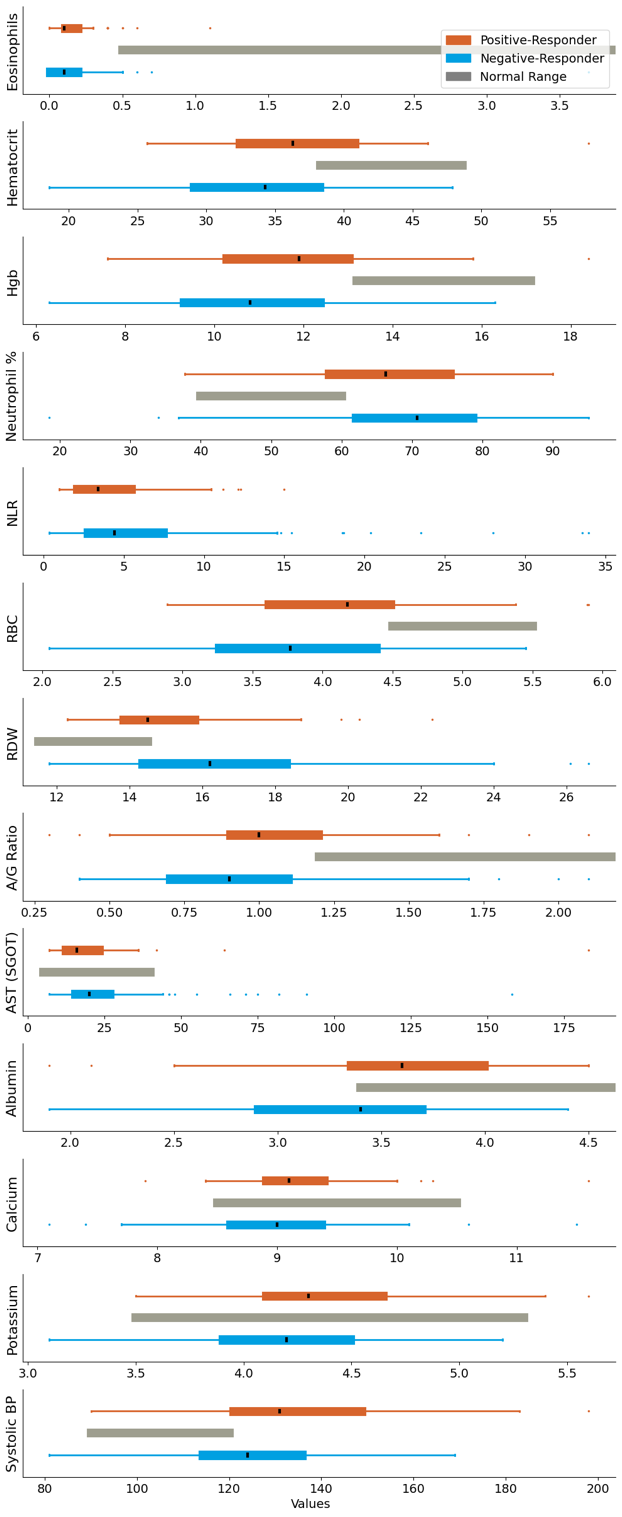


**Supplemental Figure 2. Laboratory Tests and Vital Signs Exhibiting Significant Differences Between Groups.** The red lines represent the responders, and the blue lines represent the non-responders. Dots indicate the mean values, with the length of the lines showing the standard deviation. The normal range of the NLR is not shown as it can vary depending on the laboratory and the studied population.


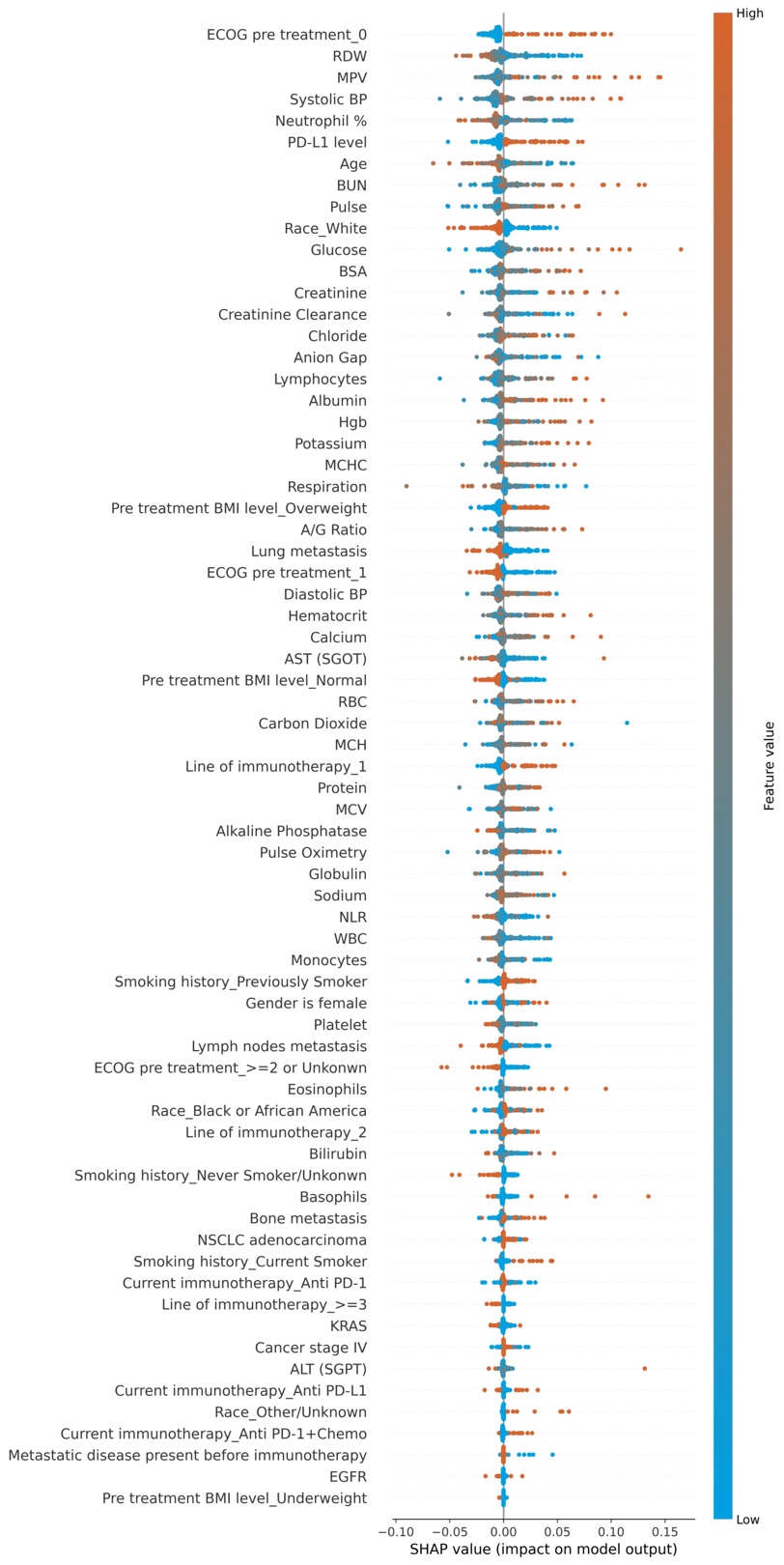


# **Supplemental Figure 3. All Features Ranked by SHAP Values.**

**
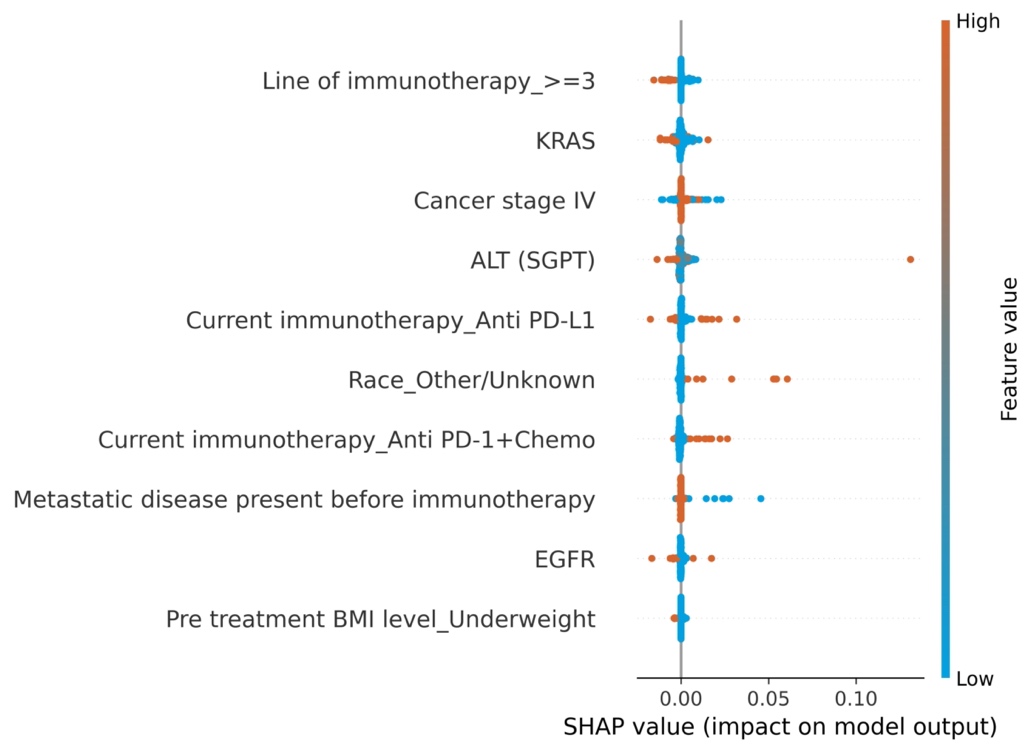
**

**Supplemental Figure 4.** **Bottom Ten Features Ranked by SHAP Value.** Each dot represents a feature’s impact on the model’s output for an individual patient. Red dots indicate higher feature values and blue dots represent lower values. The position on the x-axis shows the SHAP value, with more positive values indicating a higher likelihood of immunotherapy response. Features are ranked from top to bottom by their impact. Rankings of SHAP value for all features can be found in Supplemental Figure 2.

#

**Supplemental Figure 5. Scatter Plots for Top Features in the Prediction Model.** Each dot is a single prediction from the datase4. The x-axis is the value of the feature. The y-axis is the SHAP value for that feature, which represents how much knowing that feature’s value changes the output of the model for that sample’s prediction. A SHAP value above zero suggests that that observation is predicted as responding to the treatment by the model. Conversely, a SHAP value below zero implies no responding to the treatment. The light gray area at the bottom of the plot is a histogram showing the distribution of data values.

Supplemental Figure 5 illustrates the scatter plots for RDW, MPV, SBP, and neutrophil percentages. Scatter plots for ECOG PS and PD-L1 expression level are not presented as these variables are categorical. Supplemental Figures 4(a) and 4(d) illustrate negative correlations between the SHAP values and the increasing levels of RDW and neutrophil percentage, respectively, suggesting that patients with lower RDW values and neutrophil percentage are predicted to have better treatment responses. In contrast, Supplemental Figures 4(b) and 4(c) depict an ascending trend in SHAP values in correlation with increasing MPV and SBP, signifying that patients with elevated MPV and SBP measurements are predictive of treatment response.

**Supplemental Figure 6. AUROC Curves Comparing the Full Patient Cohort and the Subgroups of Patients Who Underwent PD-L1 or EGFR Testing.** Compared to Figure 2 and Supplemental Figure 6(a), PD-L1 shows a lower AUROC score within the subgroup of patients who underwent PD-L1 testing. Similarly, as shown in Supplemental Figures 6(b) and 6(c), EGFR exhibits AUROC scores below 0.5 in both the full patient cohort and the EGFR-tested subgroup, with the AUROC further reduced in the tested subgroup.
